# Danger of Hypodermic Injections

**Published:** 1866-06

**Authors:** 


Danger of Hypodermic Injections.—The London *Medical Times and Gazette* describes an accident which lately happened to Prof. Nassbaum, of Munich. Suffering from neuralgia, he had injected morphia under his own skin more than 2,000 times, sometimes to the extent of five grains in twenty-five hours. On one occasion lately he injected two grains acetate of morphia dissolved in fifteen minims of water into a subcutaneous vein, and for two hours his life was in imminent peril. We do not see clearly why the result should be serious, unless a small portion of air was injected. The Professor must have gone to work very carelessly, either to do this, or to penetrate a vein. Nothing is more easy than to avoid both accidents. After all, we may excuse the one mistake in 2,000 operations.—*Pacific Medical and Surgical Journal and Press.*

---

Ether.—A wealthy gentleman of Boston has placed a sum of money in the hands of an architect, for erecting a monument in that city to commemorate the discovery of the anæsthetic properties of Ether.

